# Environment-Dependent Variation in Gut Microbiota of an Oviparous Lizard (*Calotes versicolor*)

**DOI:** 10.3390/ani11082461

**Published:** 2021-08-21

**Authors:** Lin Zhang, Fang Yang, Ning Li, Buddhi Dayananda

**Affiliations:** 1 School of Basic Medical Sciences, Hubei University of Chinese Medicine, Wuhan 430065, China; 2 State Key Laboratory of Microbial Technology, Institute of Microbial Technology, Shandong University, Qingdao 266237, China; 3 School of Laboratory Medicine, Hubei University of Chinese Medicine, Wuhan 430065, China; fangyang09@163.com; 4 College of Food Science, Nanjing Xiaozhuang University, Nanjing 211171, China; lining196@126.com; 5 School of Agriculture and Food Sciences, The University of Queensland, Brisbane, QLD 4072, Australia; b.dayananda@uq.edu.au

**Keywords:** *Calotes versicolor*, gut microbiota, spatial heterogeneity, 16s rRNA

## Abstract

**Simple Summary:**

The different gut sections potentially provide different habitats for gut microbiota. We found that *Bacteroidetes*, *Firmicutes*, and *Proteobacteria* were the three primary phyla in gut microbiota of *C. versicolor*. The relative abundance of dominant phyla *Bacteroidetes* and *Firmicutes* exhibited an increasing trend from the small intestine to the large intestine, and there was a higher abundance of genus *Bacteroides* (Class: *Bacteroidia*), *Coprobacillus* and *Eubacterium* (Class: *Erysipelotrichia*), *Parabacteroides* (Family: *Porphyromonadaceae*) and *Ruminococcus* (Family: *Lachnospiraceae*), and Family *Odoribacteraceae* and *Rikenellaceae* in the hindgut, and some metabolic pathways were higher in the hindgut. Our results reveal the variations of gut microbiota composition and metabolic pathways in different parts of the lizards’ intestine.

**Abstract:**

Vertebrates maintain complex symbiotic relationships with microbiota living within their gastrointestinal tracts which reflects the ecological and evolutionary relationship between hosts and their gut microbiota. However, this understanding is limited in lizards and the spatial heterogeneity and co-occurrence patterns of gut microbiota inside the gastrointestinal tracts of a host and variations of microbial community among samples remain poorly understood. To address this issue and provide a guide for gut microbiota sampling from lizards, we investigated the bacteria in three gut locations of the oriental garden lizard (*Calotes versicolor*) and the data were analyzed for bacterial composition by 16S ribosomal RNA (16S rRNA) gene amplicon sequencing. We found the relative abundance of the dominant phyla exhibited an increasing trend from the small intestine to the large intestine, and phyla *Firmicutes*, *Bacteroidetes* and *Proteobacteria* were the three primary phyla in the gut microbiota of *C. versicolor*. There were a higher abundance of genus *Bacteroides* (Class: *Bacteroidia*), *Coprobacillus* and *Eubacterium* (Class: *Erysipelotrichia*), *Parabacteroides* (Family: *Porphyromonadaceae*) and *Ruminococcus* (Family: *Lachnospiraceae*), and Family *Odoribacteraceae* and *Rikenellaceae* in the sample from the hindgut. The secondary bile acid biosynthesis, glycosaminoglycan degradation, sphingolipid metabolism and lysosome were significantly higher in the hindgut than that in the small intestine. Taken together our results indicate variations of gut microbiota composition and metabolic pathway in different parts of the oriental garden lizard.

## 1. Introduction

The gut is the primary site for absorbing and reformulating nutrients from the food [1,2]. Microbiota of the vertebrate gastrointestinal tract have complex symbiotic relationships with their host. The gut microbial community structure significantly influences the host ecology and evolution via energy budget [3], foraging behavior [4], immunity [5,6,7], nutrient metabolism [8,9], and reproductive performance [10,11]. However, the composition of the gut microbial community is influenced by environmental factors, pH, oxygen concentration, nutrient composition, and other physiological characteristics in different gut chambers [12,13,14,15,16,17]. To date most microbiota and their relationship with host studies have focused on invertebrates [18,19], fish [20,21,22,23], amphibians [24,25,26], birds [15,27,28], mammals [29,30,31] and some reptiles [2,11,13,32]. However, studies on lizards’ gut microbial ecology and their relationship with the host are very limited. The lizrads’ gut microbial community compositions and structures were similar to those observed in mammals, and previous studies have documented general patterns. Further, gut bacterial diversity did not depend on the diversification of lizard hosts [33], but it varied along altitudes, diet and captive environment [2,14,34,35,36,37,38], and changed due to climate warming [32]. There was no significant difference in gut bacterial diversity between juveniles and adults [35], but males had significantly higher gut bacterial diversity and richness than do females [39], while non-gestation females had higher gut bacterial richness than do late-gravid females [11].

There is a complex gut microbiota composition that varies between different regions in the gastrointestinal tract [40]. Physiological changes in different areas of the small intestine and the large intestine, including chemical and nutritional gradients and isolated host immune activity, are thought to affect the composition of bacterial communities [41]. The microbiome composition differs between the large intestine and small intestine in *Agkistrodon piscivorus* [42]. However, the differences in the relative abundance in *Shinisaurus crocodilurus* were reported, such as *Bacteroidetes* (32.1%) and *Proteobacteria* (47.9%) were the dominant phyla in the cloacal swab samples [38], whereas *Firmicutes* (61.2%) and *Proteobacteria* (35.8%) were the dominant phyla in fecal samples [35]. The majority of these studies have been conducted using fecal samples, cloaca swabs, or the intestinal contents collected from wild-caught or captive individuals. Microbiota plays an important role in host physiology, such as in nutrient digestion and uptake, and in the synthesis of fatty acids, amino acids and vitamins [43,44]. The microbial metabolites include short chain fatty acids from bacterial degradation of dietary fiber [45], secondary bile acids originating from the bacterial conversion of bile acids in the colon [46], and the product of microbial-host co-metabolism of nutrients [45] protect host health [47]. There are differences in metabolic production in different intestines basing on the bacterial community compositions. However, this understanding is limited in lizards and the spatial variations and co-occurrence patterns of gut microbiota inside the gastrointestinal tracts of a host and variations of microbial community among samples remain poorly understood.

To address this issue from lizards and provide a guide for gut microbiota sampling from lizards, we investigated the bacteria in three gut locations and microbiota composition of the oriental garden lizard (*Calotes versicolor*) and analyzed using 16S ribosomal RNA (16S rRNA) gene amplicon sequencing. Considering the other studies [13,14,15,16], we hypothesize that (1) the microbial community composition appears to be different between the small intestine and hindgut, and (2) the difference of metabolic pathway is associated with the bacterial community.

## 2. Materials and Methods

### 2.1. Ethics Statement

All experiments, including the sample collection, complied with the current laws of China for the care and use of experimental animals, and followed the principles of the Ethical Committee for Experimental Animal Welfare of the Hangzhou Normal University (No. 2018135).

### 2.2. Sample Collection

We collected healthy and non-pregnant *C. versicolor* females from Hainan, China in June 2019. Then, we selected nine lizards (no significant difference in body mass) that were transported to the lab and raised under the same conditions for one week to experiment, with water and foods with the vitamin and minerals. To get the sample from living individuals, hindgut contents were collected from the large intestine (AI) as described in a previous study [48]: Firstly, the animal was kept in a suitable environment and a stable position, and secondly, the cloaca was cleaned using 70% ethanol. Thirdly, a sterile soft plastic tube 2 mm in diameter was inserted through the anus and allowed to travel about 1 cm into the intestine; fourthly, the tube was removed, along with any fecal material collected; and finally, the tube was cut into sections. Sections that did not contain fecal material were discarded and sections containing fecal material were placed in a sterilized 1.5 mL Eppendorf tube. Then we killed the nine lizards by declaration, dissected out the whole intestine from them, and defined two regions as small intestine (SI) and large intestine (LI), and then the entire contents in each region were gently squeezed out and harvested separately. Finally, 27 samples were stored at −80 °C in the laboratory for microbiota analysis.

### 2.3. DNA Extraction and Amplification

Total DNA was extracted using the cetyltrimethylammonium bromide (CTAB)/sodium dodecyl sulfate (SDS) method. Universal primer pairs 338*F* (5′-ACTCCTACGGGAGGCAGCA-3′) and 806*R* (5′-GGACTACHVGGGTWTCTAAT-3′) were used to amplify the V3-V4 region of the bacteria 16S rRNA gene using GeneAmp 2720 (ABI, Foster City, CA, USA). The 5′- end of forward primers harbors 7–12 bp unique barcodes, which were used to split each sample. All samples were mixed with an equal molar amount from the purified PCR product of each sample, and library was prepared using the TruSeq Nano DNA LT Library Prep Kit (Illumina, Sangon Biotech Co., Ltd., Shanghai, China). The purified library was sequenced using a MiSeq Reagent Kit V3 (Illumina, Sangon Biotech Co., Ltd., Shanghai, China) with an Illumina MiSeq platform (San Diego, CA, USA) to sequence according to the Wuhan Frasergen Bio-pharm Technology (Wuhan, Hubei, China).

### 2.4. Sequence Analyses

We used the QIIME2 software package (https://qiime2.org/, accessed on 24 November 2020) to process and analysis the raw reads [49]. According to the unique barcodes, sequences were identified and allocated to each sample. To get the unique amplicon sequence variant (ASV) which can be thought of as 100% operational taxonomic unit (OTUs), quality control, merging, filtering and removing low-quality sequences (reads length < 30 bp, with ambiguous base ‘N’, and average base quality score < 30) were performed using Divisive Amplicon Denoising Algorithm 2 [50]. To identify taxonomically, we employed the classify-sklearn function in QIIME2 to blast sequences against the Greengenes database (Release 13.8; http://greengenes.secondgenome.com/, accessed on 20 October 2020) [51] and the Silva database (Release 132; http://www.arb-silva.de, accessed on 20 October 2020) [52].

### 2.5. Statistical Analyses

Alpha-diversity index (the Chao1 index, the Observed species number, the Shannon index, the Simpson index, the Good’s coverage, the Faith’s PD, and the Pielou’s evenness) was calculated by *vegan* [53] and *picante* [54] package in R version 4.0.4 [55]. The Shannon–Wiener index curve is a sufficient amount of OTUs were detected and leveled off generally at sequencing depth, and Good’s coverage estimation indicates the sample size we have sequenced is representative of the bacterial microbiota. The one-way ANOVA was performed to detect variations in alpha diversity indices among three groups. For the beta diversity metrics, principal coordinate analysis (PCoA) and analysis of similarities (ANOSIM) were used to determine the communities and structure of the gut microbiota among three groups, based on the weighted UniFrac distances in *vegan* package in R version 4.0.4. The linear discriminant analysis effect size (LEfSe) method was employed to obtain variations in microbial communities based on linear discriminant analysis (LDA) sources [56].

PICRUSt was also employed to predict the potential gene profiles from 16S rRNA gene sequencing, which allowed for the identification of several functional KEGG categories and pathways expressed. The PICRUSt2 (https://github.com/picrust/picrust2, accessed on 24 November 2020) was used to predict all ASVs based on the Kyoto Encyclopedia of Genes and Genomes (KEGG) database with the *E* value < 1 × 10 ^−5^ [57]. These genes were assigned to KEGG pathways [58] and the relative abundance in each group was calculated. The unique and shared genes between populations were also plotted in the Venn diagram and a heatmap was used to visualize genes with high relative abundance. We identified the difference of KEGG pathways at the third level for both groups by the *fitFeatureModel* function in the *metagenomeSeq* package [59].

## 3. Results

### 3.1. Bacterial Community Compositions

A total of 497,643, 484,884 and 497,237 raw reads were obtained from SI, AI and LI groups, respectively. The Shannon–Wiener index curve for all samples showed suggesting that there were sufficient sequences for further analyses (Figure S1A). Furthermore, the Good’s coverage estimation minimum values were >99.9%, indicating that most gut bacterial communities of diverse species were retrieved from all samples (Figure S1B).

**Figure 1:**
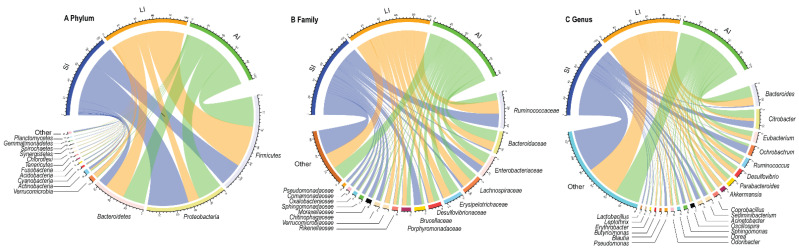
Composition of the gut microbiota of each group at the phylum (**A**), family (**B**) and genus (**C**) levels. SI: sample from the small intestine, LI: sample from the large intestine, and AI: sample from the large intestine when individuals are free-living. The visualization was prepared with R package *circlize*.

At the phylum level, *Proteobacteria* (31.81%), *Firmicutes* (39.41%), *Bacteroidetes* (21.13%), *Actinobacteria* (1.86%) were four identified dominant phyla (mean relative abundance > 1%), which contributed more than 94% of abundance across all samples (Figure 1A). At the family level, the top 17 families were listed (Figure 1B). For all samples, *Ruminococcaceae* (14.54%), *Bacteroidaceae* (9.09%) and *Enterobacteriaceae* (9.00%) were the dominant families (mean relative abundance > 9.00%), but *Brucellaceae* was more than 9.00% in SI group, *Lachnospiraceae* and *Erysipelotrichaceae* were more than 9% in LI group and AI group. At the genus level, *Bacteroides*, *Citrobacter*, *Eubacterium*, *Ochrobactrum*, *Parabacteroides*, *Akkermansia*, *Coprobacillus*, *Sediminibacterium*, *Acinetobacter* (mean relative abundance > 2%) genera were consistently present in each group (Figure 1C).

The relative abundances of plylum *Firmicutes* (*t* = −2.93, *df* = 8, *p* < 0.05) and plylum *Bacteroidetes* (*t* = −2.37, *df* = 8, *p* < 0.05) showed an increasing trend from small intestine to large intestine, but plylum *Proteobacteria* (*t* = 4.40, *df* = 8, *p* < 0.01) showed a decreasing trend from small intestine to large intestine (Figure 1A and Figure S2). Furthermore, LI and AI groups tended to have more genus *Bacteroides* (*t* = −3.04, *df* = 8, *p* < 0.05 for LI; *t* = −2.73, *df* = 8, *p* < 0.05 for AI), but less genus *Ochrobactrum* (*t* = 4.95, *df* = 8, *p* < 0.01 for LI; *t* = 3.13, *df* = 8, *p* < 0.05 for AI) than SI samples (Figure 1C).

The alpha diversities were employed to evaluate the diversity differences in the gut microbial community among the three groups (Table 1). No significant differences were detected in Chao1, the Observed species number, Shannon, Simpson, Pielou’s E, and Good’s coverage (all *p* > 0.05) upon one-way Anova except Faith’s PD, that SI had the lowest measurement of phylogenetic diversity.

**Figure 2:**
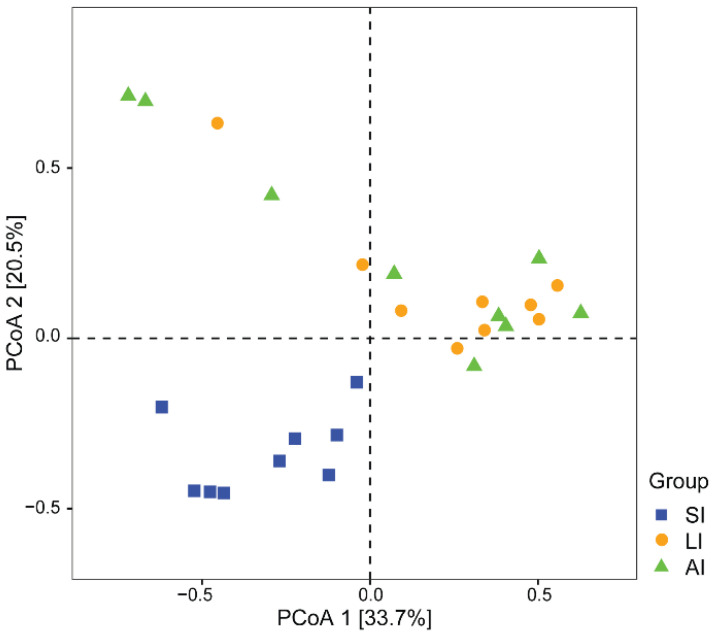
The beta diversity of the gut microbiota composition of three groups by PCoA. The variation explanation is indicated on each respective axis. SI: sample from the small intestine, LI: sample from the large intestine, and AI: sample from the large intestine when individuals are free-living.

With regard to beta diversity, the results of the PCoA plot (Figure 2) and ANOSIM showed significant differences between the SI group and other groups, respectively (SI-LI, *R* = 0.84, *p* < 0.01; SI-AI, *R* = 0.41, *p* < 0.01), with similarity between LI group and AI group (*R* = 0.02, *p* = 0.534).

### 3.2. LEfSe Analysis of Bacterial Communities

**Figure 3:**
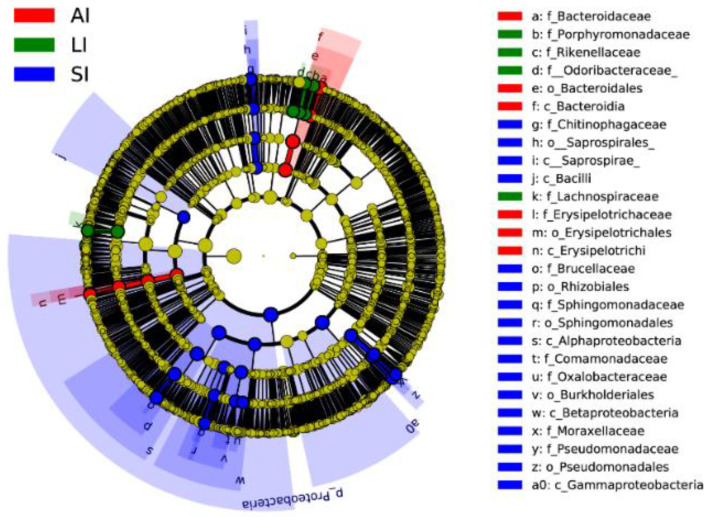
Linear discriminative analysis of effect size (LEfSe) analysis of taxonomic biomarkers of gut microbiota. Cladogram of significant changes at all taxonomic levels. The root of the cladogram represents the domain bacteria. The size of the node represents the abundance of taxa. LDA scores > 4 were shown. SI: sample from the small intestine, LI: sample from the large intestine, and AI: sample from the large intestine when individuals are free-living.

Forty biomarkers were significantly different (LDA > 4.0, *p* < 0.05), of which 23 biomarkers in the SI group were higher, 8 biomarkers in the LI group were higher, and 9 biomarkers in the AI group were higher than the other two groups based on the LDA scores (>4.0), respectively (Figure 3). Compared to SI, the AI had a higher abundance of the genus *Bacteroides* (Class: *Bacteroidia*), *Coprobacillus* and *Eubacterium* (Class: *Erysipelotrichia*), and the LI had a higher abundance of the genus *Parabacteroides* (Family: *Porphyromonadaceae*) and *Ruminococcus* (Family: *Lachnospiraceae*), and Families: *Odoribacteraceae* and *Rikenellaceae*. However, SI had a higher abundance of the genus *Acinetobacter* and *Pseudomonas* (Class: *Gammaproteobacteria*), *Sediminibacterium* (Class: *Chitinophagia*), and *Ochrobactrum* and *Sphingomonas* (phylum: *Proteobacteria*), *Comamonadaceae* and *Oxalobacteraceae* (Class: *Betaproteobacteria*) than that in the other two groups (Figure 3).

### 3.3. Functional Predictions of Bacterial

**Figure 4:**
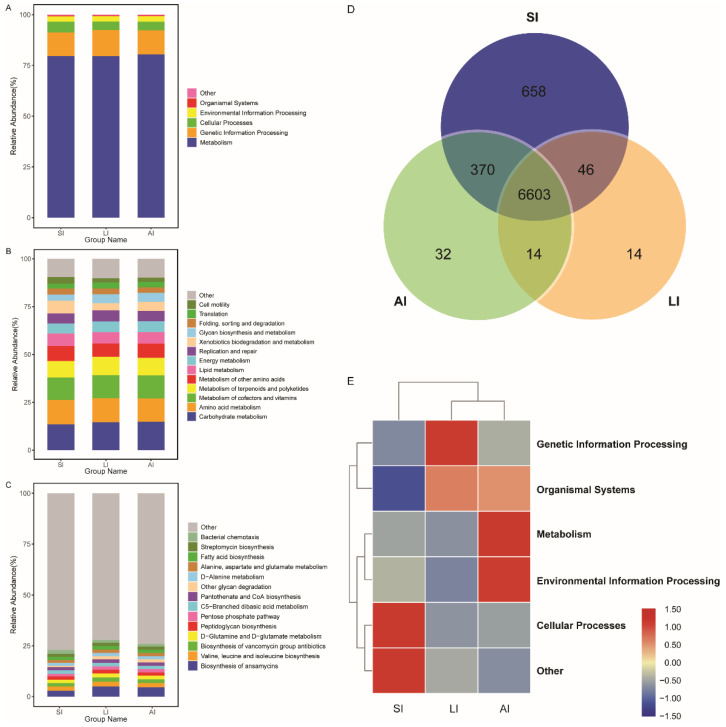
Functional classifications of 16s RNA in microbiota at (**A**) the top level, (**B**) the second level, and (**C**) the third levels of relative abundance, and (**D**) Venn and (**E**) clusters analysis of functions among three groups. SI: sample from the small intestine, LI: sample from the large intestine, and AI: sample from the large intestine when individuals are free-living.

All bacterial Amplicon Sequence Variant (ASV) species possessed Nearest Sequenced Taxon Index (NSTI) values < 2 (range from ~0.00 to 1.76). At the top level, 182 KEGG metabolic pathways were identified as metabolism (79.84%), genetic information processing (12.19%), cellular processes (4.54%), environmental information processing (2.65%), organismal systems (0.41%, Figure 4A) and other (0.38%). At the second level, 35 functions were identified, including (top 10) carbohydrate metabolism, amino acid metabolism, metabolism of cofactors and vitamins, metabolism of terpenoids and polyketides, metabolism of other amino acids, lipid metabolism, energy metabolism, replication and repair, xenobiotics biodegradation and metabolism and glycan biosynthesis and metabolism (Figure 4B), while at the third level, the biosynthesis of ansamycins was a primary function (mean relative abundance > 2%, Figure 4C).

**Figure 5:**
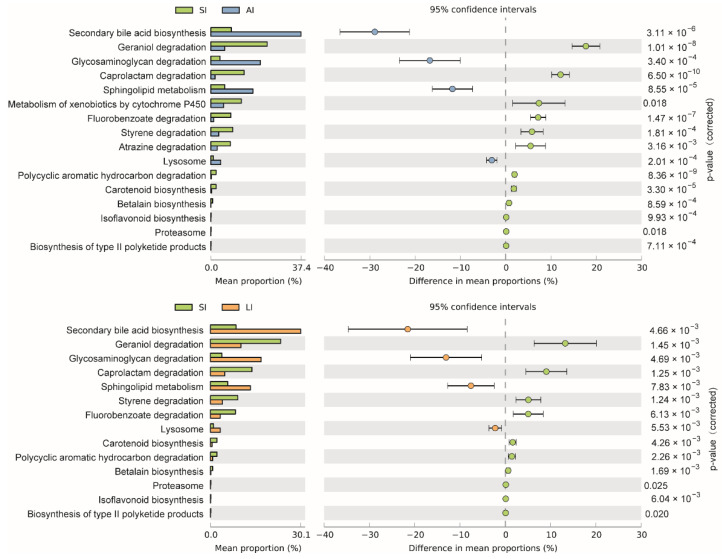
Different KEGG pathways at the third level between groups. SI: sample from the small intestine, LI: sample from the large intestine, and AI: sample from the large intestine when individuals are free-living.

The shared genes indicated that most of the knockouts (KOs) were common among the three groups, while 658, 14 and 32 KOs were exclusive to the SI, LI and AI groups, respectively (Figure 4D). The heatmap of the cluster indicated that at the top level, the KOs of SI group were enriched in Cellular processes (Figure 4E). There were significant differences at 16 pathways between SI and LI group (adj *p* < 0.05), and at 14 pathways between SI and AI group (adj *p* < 0.05), especially, secondary bile acid biosynthesis, glycosaminoglycan degradation, sphingolipid metabolism and lysosome, the four functions were higher in AI and LI group (Figure 5). There were no significant differences between AI and LI groups (all adj *p* > 0.05).

## 4. Discussion

The gut microbiota, a complex network of bacteria, fungi, protists, archaea and viruses plays a crucial role in the health of the host [2,11]. In general, the vertebrates’ gastrointestinal tract harbors a conservative bacterial assemblage dominated by *Bacteroidetes*, *Firmicutes* and *Proteobacteria* [60]. In lizards, the Phylum *Bacteroidetes* (4.2–29.1%), *Firmicutes* (2.6–81.1%) and *Proteobacteria* (1.4–85.0%) have been identified as the dominant gut microbiota [2,14,33,34,35,36,37,38,61]. In this study, *Proteobacteria* (31.81%), *Firmicutes* (39.41%), *Bacteroidetes* (21.13%) and *Actinobacteria* (1.86%) were identified as four dominant phyla, which contributed more than 94% of abundance across all samples (Figure 1A). However, the community composition is a significantly different as the hindgut samples tended to have more *Firmicutes* and *Bacteroidetes* and less *Proteobacteria* than those from the small intestine in *C. versicolor*. The difference may be resulting from the digestion status of individuals. *Bacteroidetes* was identified as the dominant phylum from fasting 30 days individuals, whereas *Firmicutes* was the dominate the dominant phylum from post-feeding individuals in *Python molurus* [62].

The small intestine (with a primary role of absorbing nutrients from food) provides a more challenging environment for bacteria with a faster flow rate and lower pH, while in comparison, the larger intestine (with a primary function to absorb water and salt from ingested material) provides a more stable environment with mild pH and slower flow rates [14]. There was no significant difference in alpha diversity among SI, LI and AI (Table 1), but there were significant differences in beta diversity between the SI and other groups, respectively, while there is a similarity in beta diversity between LI and AI (Figure 2). Those results indicated that the microbial species and proportion were similar to each group, but the microbial composition is different between SI and other groups, and in AI is similar to LI, which is consistent with previous studies that the relative abundance of dominant phyla *Bacteroidetes* and *Firmicutes* exhibited an increasing trend from small intestine to large intestine in vertebrates [13,14,15,16,17,63]. The biochemical properties of gut chambers of the intestinal tract depend on pH, nutrient composition and other characteristics [12], which may impact the microbial community structure. There was a higher relative abundance of anaerobic bacteria and facultative anaerobes bacteria in the small intestine than that in the large intestine as genus *Acinetobacter*, *Pseudomonas*, *Ochrobactrum* and *Sphingomonas* (phylum: *Proteobacteria*) (Figure 3). The genus *Acinetobacter* is associated with the immune regulation that it into autoimmunity against myelin [64], and *Acinetobacter calcoaceticus* encode peptides that mimic the amino acid sequences of myelin [65]; *Proteobacteria* contribute to the cellulose activity, degrade a variety of aromatic compounds, and boosts the nutrient absorption of their host [9].

There were higher abundances of Bacteroides (Class: Bacteroidia), Coprobacillus and Eubacterium (Order: Erysipelotrichales), Parabacteroides (Family: Porphyromonadaceae), Ruminococcus (Family: Lachnospiraceae) and Family Odoribacteraceae and Rikenellaceae in hindgut samples than those in the small intestine samples. These taxa have also been observed to assist in the maintenance of the host gut physiology, including the production of short-chain fatty acids [66,67]. Bacteroides and Parabacteroides were the most abundant genera in the gastrointestinal tract and feces in birds, mammals, reptiles and insects [2]. Bacteroides participate in the degradation of biopolymers, mainly polysaccharides [68], which are important in fermenting soluble carbohydrates in the human large intestine [69], Bacteroidetes are degrade carbohydrates and proteins in the human large intestine [70,71]. Lachnospiraceae has been demonstrated to be related to the production of butyrate, which is necessary to sustain the health of colonic epithelial tissue [72].

For encoding the energy metabolism-related enzymes, *Firmicutes* helps its host digest and absorb nutrients [73]. More exclusive KOs were found in the small intestines (Figure 4D), but the abundance of 16 KEGG pathways at the third levels were significant differences between large intestines and small intestines; there was a similar trend between AI and SI. Secondary bile acid is one of the major types of bacterial metabolites in the colon [74], gut bacteria expressing bile salt hydrolase include species in the genera *Bacteroides*, *Bifidobacterium*, *Lactobacillus*, and *Clostridium* [75]. Sphigolipid, are produced by the phylum *Bacteroidetes* (genera *Bacteroides*, *Parabacteroides*, *Prevotella*, *Porphyromonas*, *Flectobacillus*) and the *Chlorobi* (genera *Chlorobium*) [76]. Associated with the microbial composition and the function categories, the results indicated the co-evolution between a function and microbial compositions that the hindgut have more *Firmicutes* and *Bacteroidetes* and less *Proteobacteria* than those of the small intestine in *C. versicolor*. Even though some functional categories of genes of gut microbiota were found in this study, further studies are required to identify the association between the function and the gut microbiota community/assemblage. Moreover, to understand the function underlying gut microbiota, further studies exploring the genome or metagenome are required.

## 5. Conclusions

We identified significant community composition variation in the microbiota of small and large intestines in an oviparous lizard. The relative abundance of the dominant phyla exhibited an increasing trend from the small intestine to the large intestine, especially in the hindgut samples which tended to have more *Firmicutes* and *Bacteroidetes* and less *Proteobacteria* than those of the small intestine in *C. versicolor*. The difference of metabolic pathway is associated with the bacterial community, especially secondary bile acid biosynthesis, glycosaminoglycan degradation, sphingolipid metabolism and lysosome which were significantly higher in the hindgut than that in the small intestine.

## Figures and Tables

**Table 1:** The alpha diversity of microbiota among three groups in *Calotes versicolor*.

Species	SI	LI	AI	One-Way Anova
Chao1	695.22 ± 21.47	805.21 ± 25.71	866.02 ± 25.07	*F*_2,24_ = 2.155, *p* = 0.138
Observed species	691.04 ± 20.93	762.60 ± 15.03	799.51 ± 40.77	*F*_2,24_ = 2.327, *p* = 0.119
Shannon	0.92 ± 0.02	0.93 ± 0.01	0.94 ± 0.01	*F*_2,24_ = 0.657, *p* = 0.527
Simpson	5.98 ± 0.42	5.85 ± 0.30	6.09 ± 0.28	*F*_2,24_ = 0.216, *p* = 0.808
Pielou’s Evenness	0.59 ± 0.03	0.61 ± 0.02	0.64 ± 0.02	*F*_2,24_ = 0.161, *p* = 0.852
Good’s coverage	0.996 ± 0.001	0.997 ± 0.001	0.996 ± 0.001	*F*_2,24_ = 0.389, *p* = 0.682
Faith’s PD	56.07 ± 2.77 ^b^	81.44 ± 2.40 ^a^	83.21 ± 4.59 ^a^	*F*_2,24_ = 4.640, *p* = 0.020

Alpha diversity estimates mean ± se. SI: sample from the small intestine, LI: sample from the large intestine, and AI: sample from the large intestine when individuals are free-living. α = 0.05, a > b.

## Data Availability

The sequenced data were exported as individual fastq files and deposited in in Sequence Read Archive NCBI (https://www.ncbi.nlm.nih.gov/) under the accession code: PRJNA673584.

## Supplementary Materials

The following are available online at https://www.mdpi.com/article/10.3390/ani11082461/s1, Figure S1: Alpha diversity index curve. Shannon index curve (A) and Good’s coverage index curve (B), Figure S2: The relative abundance of phylum among three groups, Proteobacteria (A), Firmicutes (B), Bacteroidetes (C) and Actinobacteria (D). SI: sample from the small intestine, LI: sample from the large intestine, and AI: sample from the large intestine when individuals are free-living.
