# Supplementary material for: Environment-Dependent Variation in Gut Microbiota of an Oviparous Lizard (Calotes versicolor)
Source: Animals (Basel). 2021 Aug 21;11(8):2461. doi: 10.3390/ani11082461 (PMC8388656; doi:10.3390/ani11082461)
Supplement: Supplementary file 1 [file animals-11-02461-s001.zip › animals-1316424-supplementary.pdf]

Supplementary Materials

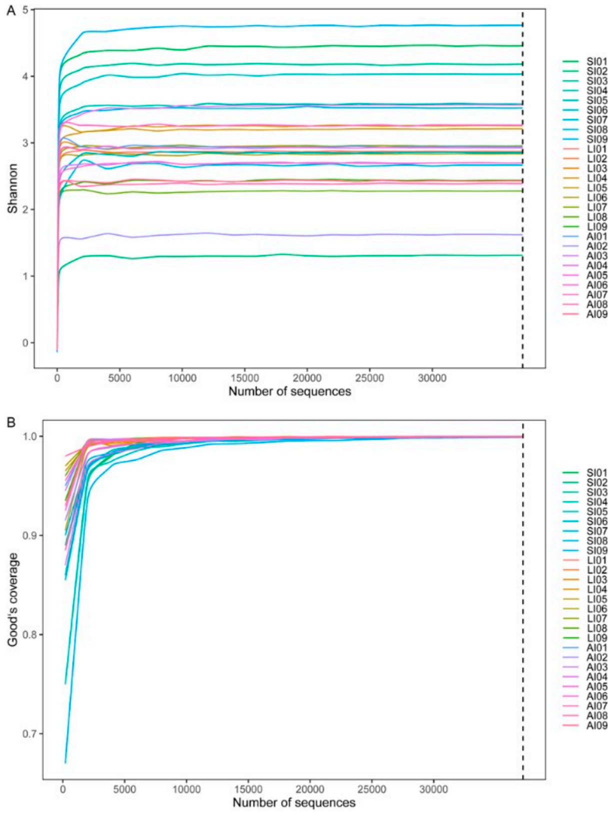

Figure S1. Alpha diversity index curve. Shannon index curve (**A**) and Good's coverage index curve (**B**).

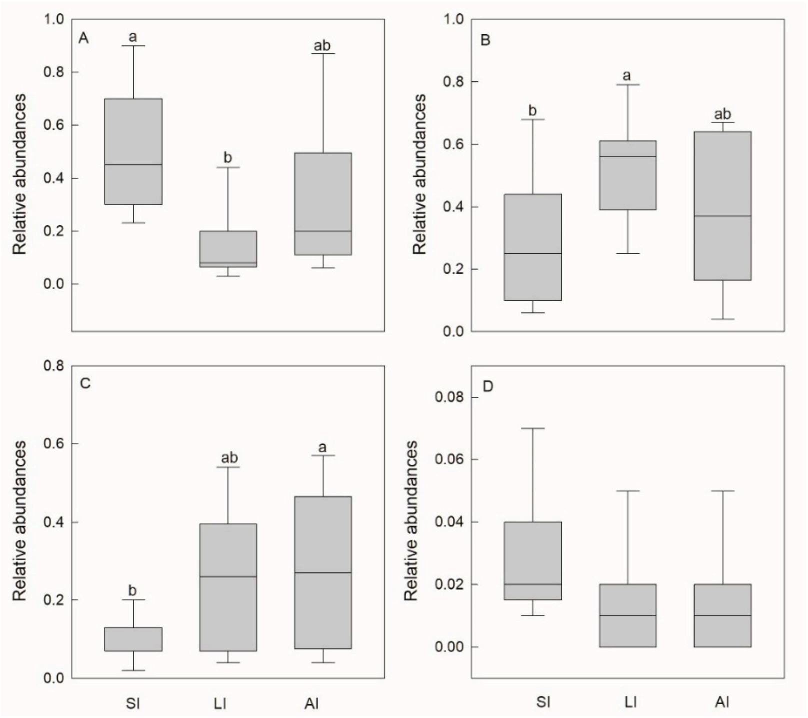

Figure S2. The relative abundance of phylum among three groups, *Proteobacteria* (**A**), *Firmicutes* (**B**), *Bacteroidetes* (**C**) and *Actinobacteria* (**D**). SI: sample from small intestine, LI: sample from large intestine, and AI: sample from large intestine when individuals are free-living.
